# Hepatitis C Viral Evolution in Genotype 1 Treatment-Naïve and Treatment-Experienced Patients Receiving Telaprevir-Based Therapy in Clinical Trials

**DOI:** 10.1371/journal.pone.0034372

**Published:** 2012-04-12

**Authors:** Tara L. Kieffer, Sandra De Meyer, Doug J. Bartels, James C. Sullivan, Eileen Z. Zhang, Ann Tigges, Inge Dierynck, Joan Spanks, Jennifer Dorrian, Min Jiang, Bambang Adiwijaya, Anne Ghys, Maria Beumont, Robert S. Kauffman, Nathalie Adda, Ira M. Jacobson, Kenneth E. Sherman, Stefan Zeuzem, Ann D. Kwong, Gaston Picchio

**Affiliations:** 1 Vertex Pharmaceuticals Incorporated, Cambridge, Massachusetts, United States of America; 2 Janssen Infectious Diseases BVBA, Beerse, Belgium; 3 Weill Cornell Medical College, New York, New York, United States of America; 4 University of Cincinnati College of Medicine, Cincinnati, Ohio, United States of America; 5 Johann Wolfgang Goethe University Medical Center, Frankfurt am Main, Germany; 6 Janssen Research and Development, Titusville, New Jersey, United States of America; Saint Louis University, United States of America

## Abstract

**Background:**

In patients with genotype 1 chronic hepatitis C infection, telaprevir (TVR) in combination with peginterferon and ribavirin (PR) significantly increased sustained virologic response (SVR) rates compared with PR alone. However, genotypic changes could be observed in TVR-treated patients who did not achieve an SVR.

**Methods:**

Population sequence analysis of the NS3•4A region was performed in patients who did not achieve SVR with TVR-based treatment.

**Results:**

Resistant variants were observed after treatment with a telaprevir-based regimen in 12% of treatment-naïve patients (ADVANCE; T12PR arm), 6% of prior relapsers, 24% of prior partial responders, and 51% of prior null responder patients (REALIZE, T12PR48 arms). NS3 protease variants V36M, R155K, and V36M+R155K emerged frequently in patients with genotype 1a and V36A, T54A, and A156S/T in patients with genotype 1b. Lower-level resistance to telaprevir was conferred by V36A/M, T54A/S, R155K/T, and A156S variants; and higher-level resistance to telaprevir was conferred by A156T and V36M+R155K variants. Virologic failure during telaprevir treatment was more common in patients with genotype 1a and in prior PR nonresponder patients and was associated with higher-level telaprevir-resistant variants. Relapse was usually associated with wild-type or lower-level resistant variants. After treatment, viral populations were wild-type with a median time of 10 months for genotype 1a and 3 weeks for genotype 1b patients.

**Conclusions:**

A consistent, subtype-dependent resistance profile was observed in patients who did not achieve an SVR with telaprevir-based treatment. The primary role of TVR is to inhibit wild-type virus and variants with lower-levels of resistance to telaprevir. The complementary role of PR is to clear any remaining telaprevir-resistant variants, especially higher-level telaprevir-resistant variants. Resistant variants are detectable in most patients who fail to achieve SVR, but their levels decline over time after treatment.

## Introduction

The hepatitis C virus (HCV) NS3•4A protease is essential for viral replication, and compounds that inhibit this enzyme represent a new class of direct acting antivirals that have been recently approved for the treatment of HCV infection [1]–[5]. Telaprevir (TVR, T) is a specific, reversible, covalent, tight- and slow-binding NS3•4A protease inhibitor [6], [7]. Results from telaprevir Phase 3 trials showed that sustained virologic response (SVR) rates were significantly higher with a regimen of 12 weeks of telaprevir in combination with either 24 or 48 weeks of peginterferon (P) and ribavirin (R) (PR), than with 48 weeks of PR alone [8], [9]. This increase in SVR rates was observed across a broad range of patient populations, including treatment-naïve patients (ADVANCE and ILLUMINATE trials) and all categories of PR-treatment-experienced patients: prior relapsers, partial, and null responders (REALIZE trial). However, in patients not responding to T/PR treatment, selection of HCV variants with decreased sensitivity to telaprevir can be observed [8]–[11], similar to other direct-acting antivirals.

HCV has higher sequence diversity, even within an individual genotype, compared with other common chronic viral infections, such as hepatitis B virus (HBV) or human immunodeficiency virus (HIV) [12]. This vast genetic diversity results from the high rate of HCV replication (with up to a trillion virions produced each day) [13] and the error-prone nature of the HCV RNA-dependent RNA polymerase (with one mutation introduced, on average, into every new genome) [14]. Indeed, it has been estimated that in an HCV-infected patient, variants with every possible single and double point mutation and even some triple mutations are created at least once each day [15], some of which exhibit varying degrees of resistance to protease inhibitors. However, these mutations may have decreased replicative fitness compared with wild-type virus, and therefore would only be present at low levels. Thus, naturally occurring protease inhibitor-resistant variants with 2 or fewer mutations are assumed to be present before treatment in all patients, and can be selected in patients with suboptimal response to treatment.

The long-term clinical implications of treatment-selected HCV variants with reduced sensitivity to telaprevir have not been established. Unlike HIV or HBV, which are both chronic infections with long-lasting nuclear DNA forms capable of archiving resistant mutations [16], [17], HCV is an RNA virus that can be eliminated and has an exclusively cytoplasmic lifecycle [18]. Therefore, no long-lived reservoir of HCV is expected, nor has one been demonstrated, allowing for the loss of less fit variants from the viral population. The rate of this loss depends on several factors, including the composition of the viral quasispecies at the time of failure and the relative fitness of the viral population containing the resistant variants. Even though in vitro data and early clinical observations have shown that telaprevir-resistant variants have a fitness disadvantage in the absence of telaprevir [10], the ability and time-frame of the viral population to return to the pre-treatment state have not been previously described.

To better understand the impact of protease inhibitor treatment on the dynamic nature of the HCV population, we analyzed viral sequences from individual patients enrolled in telaprevir clinical trials before treatment, to define the baseline prevalence of resistant variants, and during treatment in patients who failed to achieve an SVR, to define the relationship between treatment failure and emergence of resistant variants. Further analyses were performed in patients after treatment to evaluate the evolution of resistant variants in the absence of drug selective pressure. These analyses provided an understanding of factors involved in the selection of resistant variants in patients treated with telaprevir, and have been important in optimizing telaprevir treatment regimens by increasing SVR rates and minimizing clinical resistance.

## Methods

### Ethics Statement

Studies were conducted in full compliance with the guidelines of Good Clinical Practice and of the World Medical Assembly Declaration of Helsinki. Prior to study initiation, protocols and informed consent forms were reviewed and approved by institutional review boards at each site (see Appendix). All patients provided written informed consent before participating in any study-related activity.

### Patient Population

This study included treatment-naïve and treatment-experienced patients who had chronic genotype 1 HCV infection and were enrolled in Phase 2 [19]–[23] and Phase 3 clinical studies of telaprevir [8], [9], [24]. In Phase 3 clinical trials, all patients received 8 or 12 weeks of telaprevir-based treatment followed by an additional PR phase for a total treatment duration of either 24 or 48 weeks as determined by response-guided design. Treatment-experienced patients were categorized by their previous response to PR therapy: null responders, partial responders, or relapsers. Null responders exhibited a reduction of less than 2 log_10_ in HCV RNA after 12 weeks of PR therapy. Partial responders exhibited a reduction in 2 log_10_ or more in HCV RNA after 12 weeks of PR therapy but never achieved undetectable HCV RNA. Relapsers exhibited undetectable HCV RNA at the end of a previous course of PR therapy with detectable HCV RNA thereafter. Where indicated, null responders and partial responders were collectively referred to as non-responders. Further details on the study designs can be found in Jacobson et al., 2011 (ClinicalTrials.gov number NCT00627926); Zeuzem et al., 2011 (NCT00703118), and Sherman et al., 2011 (NCT00758043).

Clinical virology studies were performed in the subset of patients who had not achieved an SVR to help elucidate the reason for and consequence of treatment failure. Treatment failure was categorized as either on-treatment virologic failure or relapse.

### Definition of Treatment Outcomes

In patients who did not achieve an SVR, treatment outcomes were categorized as on-treatment virologic failure, relapse, or other. On-treatment virologic failure included patients who met a protocol-defined virologic stopping rule or patients with viral breakthrough. Viral breakthrough was defined as a confirmed on-treatment increase in HCV RNA levels of 1-log_10_ above nadir, or greater than 100 IU/mL in patients who previously had undetectable HCV RNA or HCV RNA levels below 25 IU/mL (for Study C208 and REALIZE). On-treatment virologic failure was further categorized based on its occurrence during the telaprevir combination treatment phase versus the PR treatment phase. Relapse was calculated based on the number of patients with HCV RNA below 25 IU/mL at the end of planned treatment followed by HCV RNA levels above or equal to 25 IU/mL after the end of planned treatment. Patients with missing SVR assessment and patients with HCV RNA>25 IU/mL but no viral breakthrough at planned end of treatment were categorized as “other”.

### HCV RNA Quantitation and Subtyping

Plasma HCV RNA levels were determined using the Roche COBAS TaqMan® HCV/HPS assay (Version 2.0) for Phase 3 trials. The lower limit of quantification (LLOQ) was 25 IU/mL. Results below the LLOQ were reported as “<25, target detected” (or <25 detectable HCV RNA), or “<25, target not detected” (or undetectable HCV RNA). HCV genotype/subtype was determined by sequence analysis of the HCV NS3•4A region.

### HCV RNA Sequencing

Population sequence analysis (sensitivity ∼20%) of the NS3•4A region was performed for samples with HCV RNA levels above the limit of detection (LOD) of the sequencing assay (1000 IU/mL) at baseline, and in patients who did not achieve SVR. Sequencing methods have been presented elsewhere [25]. Briefly, a blood sample was collected from patients by venipuncture of a forearm vein into tubes containing EDTA (K2) anticoagulant. Plasma was separated by centrifugation, aliquoted, and stored at −80°C. Sequence analysis of HCV utilized nested reverse-transcriptase polymerase chain reaction (RT-PCR) amplification of an approximately 9 kb HCV RNA fragment spanning the HCV polyprotein coding region. The resulting DNA was purified using the QIAquick 96 PCR Purification kit (Qiagen) and analyzed on an agarose gel. Purified DNA was sent to Beckman Coulter (Agencourt® Biosciences, Danvers, MA) for sequencing of the NS3•4A protease region. Sequencing was successful for >95% of attempted samples.

### Sequence Analysis

Sequences were aligned and analyzed for the presence of substitutions in the NS3•4A region using the software Mutation Surveyor (SoftGenetics, State College, PA). Potential resistance substitutions in the NS3•4A protease were identified using statistical analyses. This analysis utilized pooled sequencing data from all available patients who did not achieve an SVR with a telaprevir-based regimen in all Phase 2 and 3 studies. Briefly, the frequency of variants observed in the NS3•4A protease after treatment-failure was compared statistically against the expected frequency (derived from the pre-treatment time point). For the comparison, the time point considered representative of the treatment failure was the time of last dose of telaprevir after meeting criteria for a stopping rule or viral breakthrough, end of treatment time point, or time of relapse, where HCV RNA levels were above the sequencing assay LOD. Significance was calculated using either a Poisson distributed probability or a Fisher's exact test with a significance threshold of 0.05 (alpha) adjusted with a Bonferroni correction. Sequencing data were available for 521 patients with genotype 1a and 219 patients with genotype 1b at the treatment-failure time point. This large sample size allowed detection of significant variants even if they occurred only rarely in the population of patients who did not achieve an SVR with telaprevir-based treatment. In particular, D168N, which was observed in only 6 of 521 genotype 1a patients after treatment failure (1.15%) was still determined to be a TVR-failure associated variant because of its extremely rare detection prior to treatment (0.04%).

### Telaprevir IC_50_ determination in the HCV replicon cell assay

The IC_50_ value of telaprevir in genotype 1b was determined in a 48-hour assay using stable HCV replicon cells as described previously [26]. The IC_50_ value of telaprevir in genotype 1a was determined in a transient replicon assay. A sub-genomic replicon containing the G1a-H77 NS3-3′ sequence with 6 adaptive mutations (Q1067R, P1496L, V1655I, K1691R, K2040R, and S2204I) and a luciferase gene cassette under the translational control of the EMCV IRES and the poliovirus IRES, respectively, was constructed. Mutations of HCV NS3 protease were introduced into the genotype 1a replicon plasmid using PCR-based site-directed mutagenesis. Replicon RNA was generated from *Hpa*I-linearized genotype 1a replicon using T7 MEGAScript Kit (Ambion). 5 µg of replicon RNA was electroporated into Huh-7-ET-cured cells resuspended in Ingenio (Mirus, MIR 50117). Electroporated cells were resuspended in DMEM culture medium and plated on 96-well plates (Costar 3904) at 1×10^4^ cells in 100 µl. After incubation at 37°C for 24 h, the cell culture was added with 100 µl of medium containing compound with serial dilutions and allowed to grow for 3 days. The cells were lysed with cell lysis buffer (Promega E153A) and the luciferase activity was measured with the Luciferase kit (Promega E1501) using the Envision reader. The IC_50_ values were generated from dose-response curves with SoftMax Pro.

Multiple independent assays were conducted for each viral variant, and the mean and standard deviation (SD) of the replicon IC_50_ values were calculated. The fold change in sensitivity to telaprevir was calculated by dividing the mean IC_50_ for each variant by the mean IC_50_ for the wild type replicon. The range of assay variability for IC_50_ values is within 3-fold.

## Results

### Treatment Outcome in Patients from Phase 3 Telaprevir Studies

**Figure 1:**
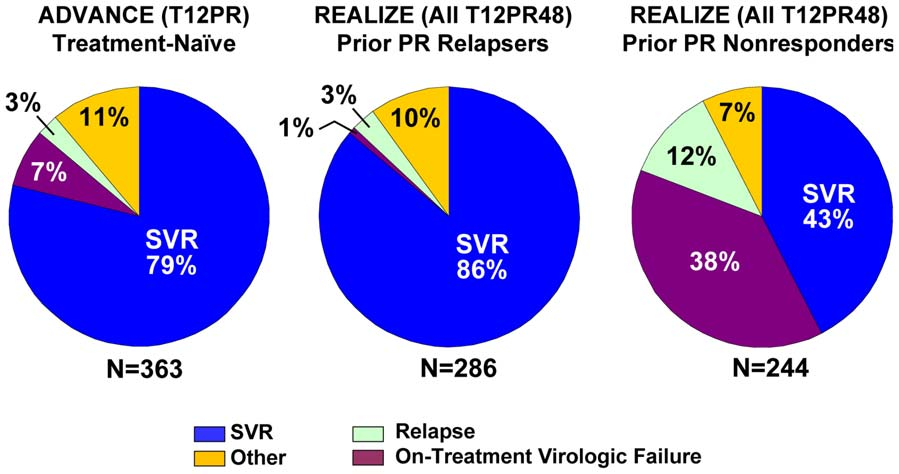
Treatment Outcome in Patients from Phase 3 Telaprevir Studies. Data from ADVANCE includes only the T12PR arm and data from REALIZE includes pooled TVR arms. ‘Other’ includes patients with missing SVR assessment and patients with HCV RNA>25 IU/mL at last study dose but who did not have viral breakthrough. ‘Relapse’ here is calculated using a denominator of total number of patients, and so differs from a relapse rate calculated in Figure 8 which uses patients with undetectable HCV RNA at the end of treatment. ‘SVR’ rates here are calculated as in the INCIVEK USPI, which utilized the last recorded HCV RNA assessment; in case of missing data, the last HCV RNA assessment from week 12 of follow-up onward was used. For the determination of SVR and relapse rates, the lower limit of quantification (<25 IU/ml) of the HCV RNA assay was used. These rates differ from SVR rates calculated according to the study protocol, which used the HCV RNA assessment at week 24 without carrying forward the prior HCV RNA data point in case of missing data, and the limit of detection (10–15 IU/ml) of the HCV RNA assay for SVR and relapse rate determination. SVR rates using the protocol analysis were: 75% for T12PR, 69% for T8PR and 44% for PR (ADVANCE, Jacobson 2011); 72%, 92% and 88% were recorded for the overall study (all patients), T12PR24 and T12PR48 randomized arms, respectively (ILLUMINATE, Sherman 2011); and 64%, 66% and 17% for T12PR48, lead-in T12PR48 and PR, respectively (REALIZE, Zeuzem 2011).

The addition of telaprevir to PR treatment significantly increased SVR rates compared to PR alone. In the treatment-naïve population (ADVANCE), 12 weeks of telaprevir response-guided combination treatment with either 24 or 48 weeks of PR resulted in a 79% SVR rate, compared with 46% in the placebo (Pbo)/PR group (*P*<0.0001) [1]. In the treatment-failure population (REALIZE), 12 weeks of telaprevir in combination with 48 weeks of PR resulted in higher SVR rates than in the Pbo/PR group: 86% compared with 22% in prior relapsers, 59% compared with 15% in prior partial responders, and 32% compared with 5% in prior null responders, (*P*<0.001) [1]. Treatment outcomes in the intent to treat (ITT) population were similar between treatment-naïve and prior relapsers with low rates of on-treatment virologic failure (7% in treatment-naïve and 1% in prior relapsers) and relapse (3% in both treatment-naïve and prior relapsers). In contrast, SVR rates were lower in patients with a prior non-response to PR as a result of higher relapse rates (12%) and much higher on-treatment virologic failure rates (38%) (Figure 1).

**Figure 2:**
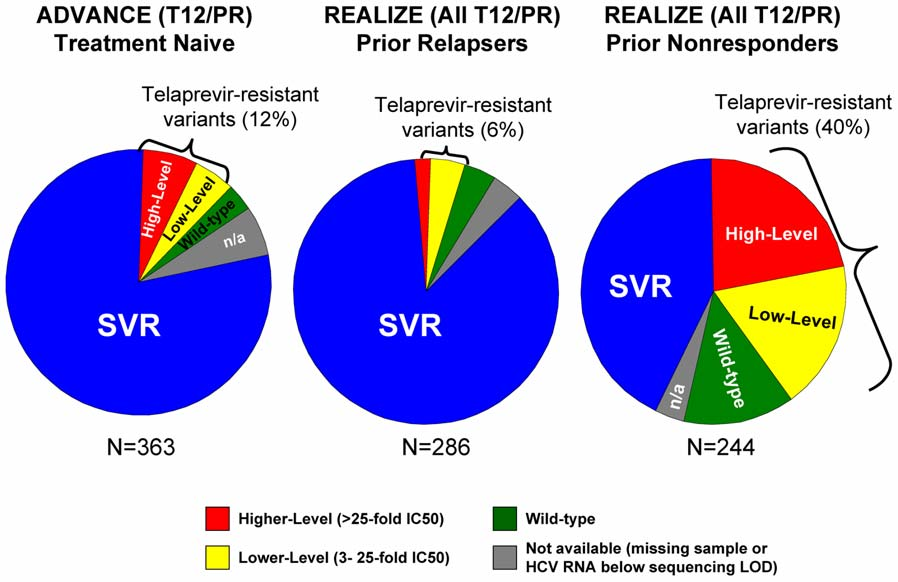
Phenotypic Resistance Profiles in Patients Who Did Not Achieve SVR with a TVR-based Regimen. Data from ADVANCE include only the T12PR arm and data from REALIZE include pooled TVR arms. Higher-level resistance (red) is defined as >25-fold increase in IC_50_ and lower-level resistance (yellow) is defined as 3- to 25-fold increase in IC_50_ from wild-type. Grey (n/a) indicates patients with no sequence data available due to HCV RNA levels below the LOD of the sequencing assay or lost-to-follow-up.

**Figure 3:**
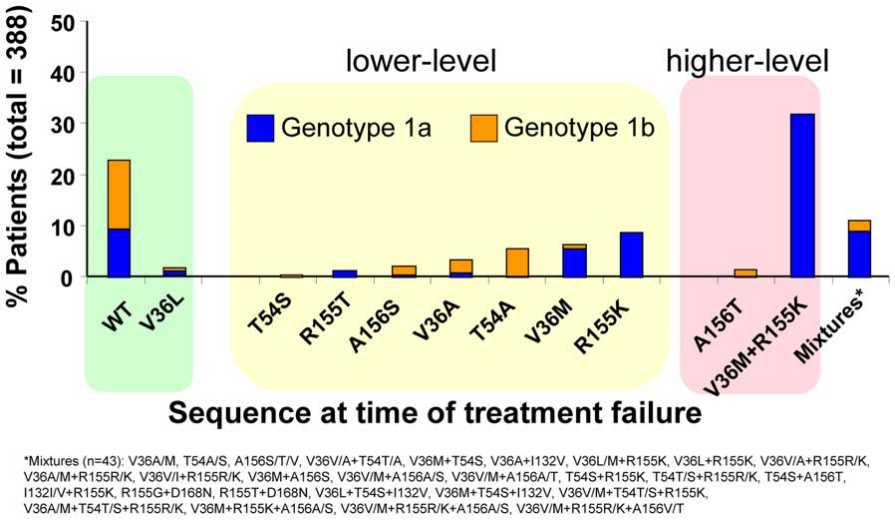
Frequency of Resistance Profiles in Patients Who Did Not Achieve an SVR with a TVR-based Regimen. X-axis is the % of patients with a given resistant variant out of all patients who did not achieve an SVR and had available sequence data in Phase 3 trials (n = 388, includes all TVR arms of all 3 Phase 3 trials). Higher-level resistance (red) is defined as >25-fold increase in IC_50_ and lower-level resistance (yellow) is defined as 3- to 25-fold increase in IC_50_ from wild-type. Variants observed in only a single subject (ie, 0.26% of the failure population) are not displayed. These variants are: V36G/I, I132V (1a), and R155M.

Resistant variants were observed in 12% (44/363) of treatment-naïve patients (ADVANCE; T12PR arm), 6% (18/286) of prior relapsers, and 40% (98/244) of prior non-responders {24% (23/97) of prior partial responders and 51% (75/147) of prior null responder patients} (REALIZE, T12PR48 arms) (Figure 2). Regardless of treatment history, the majority of patients who did not achieve SVR had detectable resistant variants at the time of treatment failure (Figure 3). The proportion of non-SVR patients with available sequence data with detectable resistant variants at the time of failure was 79% (44/56) of treatment-naïve (T12PR, ADVANCE), 62% (18/29) of prior PR relapsers, 61% (23/38) of prior PR partial responders, and 81% (75/93) of prior PR null responders (REALIZE, T12PR48 arms). In order to further understand the reason for treatment failure, virology data were analyzed by failure type in patients with on-treatment virologic failure and relapse.

### Resistance Profiles in Patients Who Did Not Achieve SVR with a Telaprevir-based Regimen in Phase 2 and 3 Studies

Telaprevir-selected variants were identified from a comprehensive analysis of patients who did not achieve SVR in both Phase 2 and 3 clinical trials; approximately 5000 unique HCV sequences were analyzed during or after treatment with telaprevir. The analysis identified the following variants as being significantly enriched in the population of patients who did not achieve SVR: V36A/M, T54A/S, R155K/T, A156S/T, and D168N (Table 1). Additionally, V36L was present at 4% after treatment failure, but was not significantly enriched due to its frequent (1.5%) occurrence at baseline (Table 1). Although not significantly enriched, variants V36G/I, I132V (subtype 1a only, subtype 1b consensus is Val at position 132), R155G/M, or A156F/N/V were observed in less than 2% of patients who did not achieve an SVR [1]. These variants are all included in the figures and analyses presented here, with the exception of V36I/L and I132V which confer less than 3-fold resistance in the replicon (see below). The amino acid positions associated with telaprevir resistance are located near the protease catalytic site in the NS3 protease domain, consistent with the mechanism of action of a protease inhibitor (Figure S1).

**Table 1:** Variants of Interest from a Pooled Analysis of Subjects Who Did Not Achieve an SVR in Phase 2 and 3 Studies.

NS3 Variant	Replicon IC_50_ Fold-Change^a^	Subtype	Treatment-Naïve Occurrence% (n)^b^	Treatment-Failure Occurrence% (n)^c^	p^d^
Significant					
R155K	5.9	1a	1.21 (27)	71.4 (372)	<1E-307
V36M	6.8	1a	0.63 (14)	63.15 (329)	<1E-307
R155T	20.0^e^	1a	0.04 (1)	3.84 (20)	8.05E-33
V36A	7.5	1a	0 (0)	2.69 (14)	6.82E-11
A156S	22.4	1a	0 (0)	1.92 (10)	5.65E-08
D168N	0.9	1a	0.04 (1)	1.15 (6)	9.52E-08
T54A	6.3	1b	0 (0)	28.31 (62)	8.55E-58
A156S	9.6	1b	0.07 (1)	10.96 (24)	3.95E-44
V36A	7.4	1b	0 (0)	16.89 (37)	7.88E-34
A156T	>62^f^	1b	0 (0)	7.31 (16)	1.00E-14
V36M	7.0	1b	0.07 (1)	3.65 (8)	6.60E-12
T54S	4.2	1b	2.11 (29)	7.31 (16)	2.77E-05
Observed but not significant					
V36G	11.3	1b	0 (0)	0.46 (1)	1.37E-01
V36I	0.3^e^	1a	0.09 (2)	0.19 (1)	3.74E-01
V36I	0.3	1b	0.29 (4)	0.91 (2)	1.34E-01
V36L	2.2^e^	1a	1.89 (42)	4.03 (21)	1.32E-03
V36L	2.2	1b	0.95 (13)	2.28 (5)	6.02E-02
I132V	1.8	1a	0.54 (12)	1.54 (8)	8.35E-03
R155G	7.4^e^	1a	0 (0)	0.19 (1)	1.90E-01
R155M	5.6^e^	1a	0 (0)	0.19 (1)	1.90E-01
A156F	>62^f^	1b	0 (0)	0.46 (1)	1.37E-01
A156N	>62^f^	1b	0 (0)	0.91 (2)	1.88E-02
A156V	>62^f^	1b	0 (0)	0.46 (1)	1.37E-01

a Replicon IC_50_ values are the mean from at least three independent experiments with fold-change relative to wild-type (WT) replicon cells. The mean (SD) IC_50_ value of telaprevir in G1b WT (mADE) replicon cells is 0.482 (0.122) µM (n = 15). In G1a WT replicon the mean (SD) IC_50_ value of telaprevir is 0.961 (0.132) µM (n = 8).

b Numerator is number of subjects that possess the given variant; denominator is total count of subjects that have sequence data available at that position.

c Treatment-Failure Occurrence indicates the number of subjects in TPR or T/P containing groups from Phase 2 and 3 studies who did not achieve an SVR that had the given variant at the treatment-failure timepoint. The denominator is the total number of subjects from Phase 2 and 3 studies who did not achieve an SVR with treatment-failure timepoint sequencing data available.

d The value tests for enrichment of the variant in the treatment-failure population (Alpha[Bonferroni corrected]: 1a, 0.0000919; 1b, 0.000102).

e Was determined in replicon 1b.

f The replicon IC_50_ is greater than 30 µM, the maximum concentration of telaprevir used in the assay.

Two different subtype-dependent pathways for developing resistance to telaprevir were observed in patients infected with genotype 1a or genotype 1b. Overall, in patients who failed to achieve SVR (N = 388; 1a, n = 269; 1b, n = 119), variants observed were wild-type (23%; V36L 2%), T54A/S (6%), V36A/M (11%), R155K/T (10%), A156S (2%), A156T (2%), and V36M+R155K (32%). In addition to V36M+R155K, 11% of patients had variants with more than one substitution (Figure 3). In patients with genotype 1a, the predominant telaprevir-resistant variants observed were V36M (8%), R155K (13%), V36M+R155K (46%), and wild-type (14%). In patients with genotype 1b, the predominant telaprevir-resistant variants observed were V36A (8%), T54A (18%), A156S/T/V (10%), and wild-type (44%).

Additionally, we sequenced the NS3-NS5B spanning region in Phase 2 studies to evaluate the potential to select compensatory mutations, and found no consistent mutations in NS3 or any of the 4 NS3•4A protease cleavage sites [27].

### Characterization of Telaprevir-Resistant Variants

**Figure 4:**
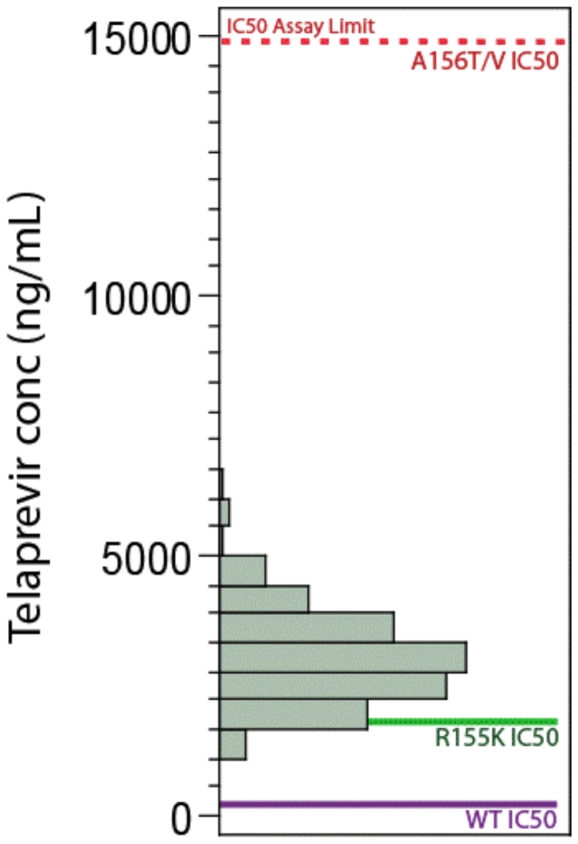
Plasma telaprevir concentration relative to the in-vitro sensitivity of wild-type and telaprevir-resistant variants. Telaprevir was dosed at 750 mg q8h.

Phenotypic studies (enzymatic and HCV replicon-based) were performed to characterize substitutions that had been identified in the HCV NS3 protease domain after treatment failure in clinical studies of telaprevir. These mutations conferred different levels of resistance to telaprevir, ranging from a 0.3- to >62-fold increase in IC_50_ from wild-type (Table 1). Variants were categorized as either lower-level resistance, defined as a 3- to 25-fold increase in IC_50_ from wild-type, or higher-level, defined as more than 25-fold increase in IC_50_. This distinction is clinically relevant, as nearly all viral breakthrough during telaprevir treatment was associated with higher-level resistant variants, indicating that telaprevir exposure was sufficient, in combination with PR, to inhibit lower-level resistant variants (Figure 4). A lower-level of resistance to telaprevir was conferred by single substitutions at V36A/G/M, T54A/S, R155G/K/M/T, and A156S. A higher-level of resistance to telaprevir was conferred by A156F/T/V and the double variant V36M+R155K. Variants V36I/L, I132V (subtype 1a only), and D168N did not change the sensitivity to telaprevir in the replicon system (conferred less than 3-fold change IC_50_). Telaprevir-resistant variants remained fully sensitive to interferon-alfa, ribavirin, and representative HCV nucleoside and non-nucleoside polymerase inhibitors in the replicon system (data not shown). Additionally, most telaprevir-resistant variants had a lower replication capacity than wild-type *in vitro* [28].

### Resistance Profiles Before Telaprevir Combination Treatment

In total, 3449 unique HCV sequences were analyzed in patients before treatment (baseline), allowing determination of the viral population sequence for over 98% of the patients enrolled in Phase 2 and Phase 3 clinical studies. Baseline resistance was detected by population sequencing in 5% of patients, most of whom had either the V36L (1.5%) or T54S (2.7%) variant, which conferred only a 2.2- or 4-fold increase in IC_50_ from wild-type, respectively. The remaining patients (less than 1%) had the more clinically-relevant V36M (0.3%), T54A (0.03%), or R155K (0.5%) mutations. All these variants conferred lower-level resistance to telaprevir, with a 6 to 7-fold change in IC_50_ from wild-type replicon. The I132V variant, which conferred a <1-fold change in IC_50_ from wild-type, was present at 0.5% of subtype 1a patients (Val is the consensus amino acid in genotype 1b). No patient had the higher-level resistant variants A156T or V36M+R155K.

To evaluate the impact of pre-existing telaprevir-resistant variants on the response to a T12PR regimen in Phase 3 trials (ADVANCE [T12 arm only], ILLUMINATE, and REALIZE), SVR rates were compared between patients with wild-type virus at baseline and patients with predominant variants (including V36L and I132V) detectable by population sequencing at baseline. The SVR rates in patients treated with T/PR were comparable, with a 70% (54/77) SVR rate for patients with variants and a 72% (964/1337) SVR rate for patients with wild-type virus at baseline. In treatment-naïve patients (ADVANCE and ILLUMINATE), the SVR rate of patients with telaprevir-resistant variants at baseline was 74% (39/53) compared with an SVR rate of 76% (634/837) in patients who did not have a variant at baseline. In treatment-failure patients (REALIZE), SVR rates in patients with telaprevir-resistant variants at baseline by prior response were 14% (1/7), 100% (2/2), and 80% (12/15) for prior null-responders, prior partial-responders, and prior relapsers, respectively, compared with SVR rates of 33% (46/140), 57% (53/93), and 87% (231/267) in patients who did not have a resistant variant at baseline.

While the overall presence of resistant variants did not appear to affect response to T/PR, an effect of individual variants was possible, in particular in patients with a poor interferon response. Therefore, the presence of the most common treatment-selected variants, V36M and R155K, were evaluated separately. Seven of the 9 treatment-naïve patients with these variants at baseline achieved an SVR, whereas neither of the 2 prior null responders with these variants at baseline achieved an SVR. These results indicate that the presence of baseline variants may impact the response to T/PR in certain patient populations, such as prior null responders.

### Resistance Profile in Patients with On-treatment Virologic Failure

**Figure 5:**
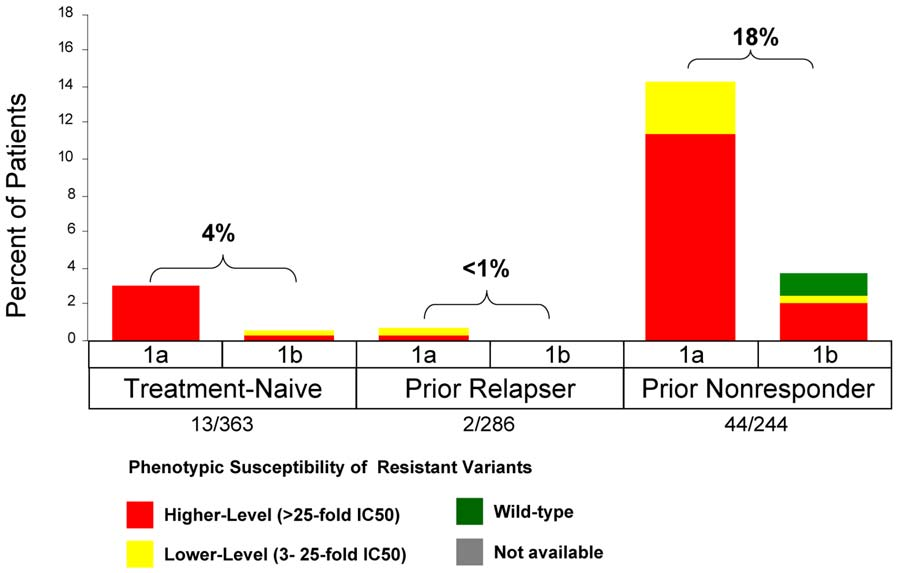
Frequency of Phenotypic Resistance Profiles in Patients with On-Treatment Virologic Failure during the TVR Treatment Phase by Prior Response and Subtype in Phase 3 Studies (includes the T12/PR arm of ADVANCE and pooled TVR arms of REALIZE). Higher-level resistance (red) is defined as >25-fold increase in IC_50_ and lower-level resistance (yellow) is defined as 3- to 25-fold increase in IC_50_ from wild-type.

**Figure 6:**
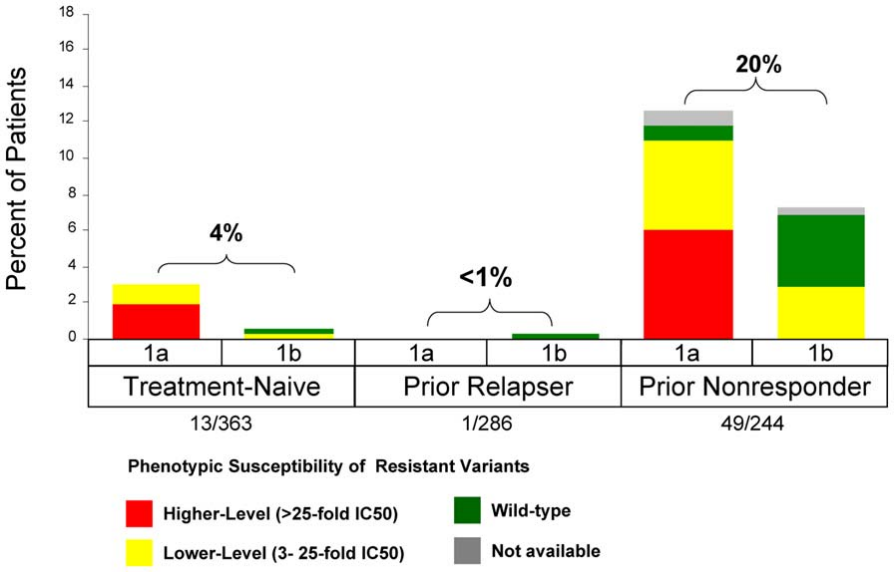
Frequency of Phenotypic Resistance Profiles in Patients with On-Treatment Virologic Failure during the PR Treatment Phase by Prior Response and Subtype in Phase 3 Studies (includes the T12/PR arm of ADVANCE and pooled TVR arms of REALIZE). Higher-level resistance (red) is defined as >25-fold increase in IC_50_ and lower-level resistance (yellow) is defined as 3- to 25-fold increase in IC_50_ from wild-type.

On-treatment virologic failure describes the outcome of patients who met a virologic stopping rule or had viral breakthrough during treatment, either during the telaprevir dosing phase or in the subsequent PR treatment phase. In Phase 3 studies, on-treatment virologic failure during the telaprevir treatment phase was consistently associated with higher-level resistance in both treatment-naïve and treatment-failure patients (Figure 5). On-treatment virologic failure was more common with genotype 1a and associated with the V36M+R155K variant. Even though a consistent resistance profile was observed across patients, differences in the rate of virologic failure varied according to the responsiveness to PR. In treatment-naïve patients (T12PR arm; ADVANCE), the on-treatment virologic failure rate was 7% (26/363), with 3.6% occurring during the telaprevir treatment phase (Week 1–12) and 3.6% during the PR treatment phase. In treatment-failure patients (T12PR48 arms; REALIZE), the on-treatment virologic failure rate was 1% (3/286) in prior relapsers and 38% (93/244) in prior non-responders to PR (18% in prior partial responders and 52% in prior null responders), with 1% and 18% during the telaprevir treatment phase. On-treatment virologic failure after telaprevir treatment, during the PR treatment phase, was associated with either wild-type virus (predominantly genotype 1b), lower- or higher-level resistant variants (Figure 6).

### Resistance Profile in Patients with Relapse

Relapse was defined as the presence of detectable HCV RNA (>25 IU/mL) during follow-up after HCV RNA<25 IU/mL at the end of planned treatment, and was calculated using as a denominator the number of patients with HCV RNA<25 IU/mL at the end of treatment. In the treatment-naïve (ADVANCE, T12PR) and prior relapser populations (REALIZE), the relapse rates were generally low in telaprevir-containing arms: 4% (11/298) and 3% (8/254), respectively (Figure 1). In the prior non-responder population, relapse rates were higher for prior partial (20%; 14/71) and prior null (24%; 15/62) responders. Relapse rates were similar between genotypes 1a and 1b.

**Figure 7:**
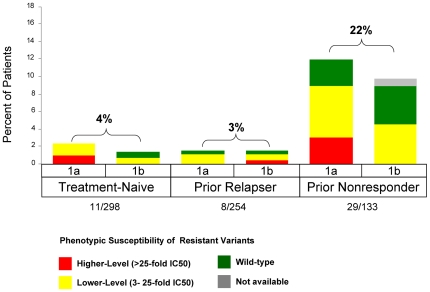
Frequency of Phenotypic Resistance Profiles in Patients who Relapse by Prior Response and Subtype in Phase 3 Studies (includes the T12/PR arm of ADVANCE and pooled TVR arms of REALIZE). Relapse was defined as HCV RNA>25 IU/mL during follow-up after <25 IU/mL at the end of planned treatment, and was calculated with a denominator of patients with undetectable HCV RNA at the end of treatment. Higher-level resistance (red) is defined as >25-fold increase in IC_50_ and lower-level resistance (yellow) is defined as 3- to 25-fold increase in IC_50_ from wild-type.

In patients who completed their assigned treatment regimen, follow-up sampling was performed 4, 12 and/or 24 weeks after the end of PR dosing. In treatment-naïve and treatment-failure populations, relapse was associated with either wild-type virus or lower-level telaprevir-resistant variants in most TPR treated patients (Figure 7). Relapse rates and resistance profiles were similar among patients who completed either 24 or 48 weeks of treatment.

### Resistance Profile in Patients Who Discontinued Treatment Early

Patients who discontinued treatment early, prior to the planned duration, for non-virologic reasons provided insight into changes in the viral population under telaprevir pressure. In general, patients who discontinued treatment early (less than 4 weeks) tended to have predominantly wild-type virus. Elimination of the wild-type viral population appeared to occur in most patients by 6 to 8 weeks of treatment. In patients who received a longer treatment duration (more than 8 weeks of telaprevir), the viral population consisted of telaprevir-resistant variants. In ADVANCE, a 12 week telaprevir duration had a higher SVR rate compared with 8 weeks, due to continued telaprevir pressure on the residual wild-type and lower-level telaprevir-resistant variants [8].

### Evolution of HCV Variants After Treatment

The evolution of resistant variants was evaluated after the end of treatment in Phase 3 studies. A conservative approach was taken in this analysis, whereby any non-wild-type variants at positions 36, 54, 155, and 156 were considered resistant, as were I132V (genotype 1a only) and D168N. Across Phase 3 trials, population sequencing revealed that 77% of 388 patients had a resistant variant following treatment failure. Of the 254 patients with resistance after treatment failure and who had additional sequencing time points, 153 no longer had detectable resistant variants within the median study follow-up period of 9.7 months.

**Figure 8:**
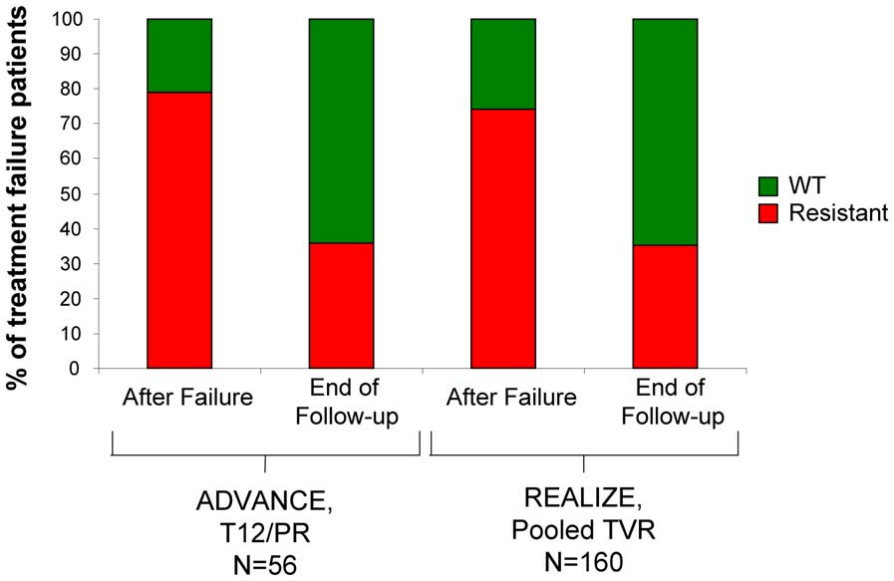
Proportion of Patients with Loss of Resistant Variants after Treatment Failure during Follow-up. Of patients with available HCV population sequence data after failing to achieve SVR, a similar fraction of treatment-naive (T12/PR arm, ADVANCE) and treatment-experienced patients (Pooled TVR Arms, REALIZE) had resistance initially after failure. ‘After Failure’ indicates resistance profile at the visit representative of treatment failure. ‘End of Follow-up’ indicates the end of the follow-up observation period (end of study), with a median time of 12 months (range: 0 to 17 months) in ADVANCE and 9 months in REALIZE (range: 0 to 17 months).

Significantly, prior treatment status (naïve versus experienced) did not affect the fraction of patients who lost resistant variants by the end of the study follow-up (Figure 8). Among treatment-naive patients in the T12 arm of ADVANCE, 44 patients had resistant variants after treatment, and in 56% of these patients only wild-type virus was detected within the follow-up observation period (median time, 12 months). Similarly, among treatment-experienced patients in REALIZE, 118 patients had resistant variants after treatment and in 53% only wild-type virus was detected within the follow-up observation period (median time, 9 months). Together, this resulted in approximately 35% of non-SVR patients with available sequence data having resistance at the end of the follow-up period, regardless of prior treatment status.

As reported in more detail in Sullivan *et al.* 2011 [29], Kaplan-Meier (KM) estimation was used to explore the rate of loss of the resistant variants. Interestingly, a significant difference was noted between genotype 1a and 1b subtypes. Based on all patients failing telaprevir combination treatment in a Phase 3 trial, the KM estimated median (95% CI) time to wild-type after treatment failure was 10.7 (9.6, 12.4) months in the case of 1a (N = 269) and only 0.9 (0.00, 2.10) months in the case of 1b (N = 119).

## Discussion

Telaprevir in combination with peginterferon and ribavirin significantly increased sustained virologic response rates compared with PR alone in Phase 3 studies of treatment-naïve and treatment-experienced genotype-1 HCV patients. Genotypic and phenotypic analyses from telaprevir clinical trials were performed to understand the relationship between virologic failure to a telaprevir-containing regimen and emergence of HCV variants with decreased sensitivity to telaprevir. In Phase 3 trials, resistant variants were observed during treatment with telaprevir in 12% of treatment-naïve patients (ADVANCE; T12PR arm), 6% of prior relapsers, 24% of prior partial responders, and 51% of prior null responder patients (REALIZE, T12PR48 arms).

Clinical virology results from this extensive dataset have established a well-characterized, consistent resistance profile for telaprevir in genotype 1 HCV-infected patients. Lower-level resistance to telaprevir (3- to 25-fold decrease in sensitivity) was conferred by V36A/M, T54A/S, R155K/T, and A156S variants. Higher-level resistance to telaprevir (over 25-fold decrease in sensitivity) was conferred by A156T and V36M+R155K variants. The predominant telaprevir-resistant variants observed in patients with subtype 1a were V36M, R155K, and V36M+R155K. In patients with subtype 1b, the predominant variants observed were V36A, T54A, and A156S/T (Figure 3). The pathway of V36M and R155K substitutions is not observed in subtype 1b most likely due to the need for 2 nucleotide changes for each substitution in this subtype compared with the need for only a single nucleotide change in subtype 1a, but other restrictions may also exist.

### Baseline Resistance

Baseline sequencing from telaprevir clinical trials suggest that patients with naturally occurring telaprevir-resistant variants are uncommon (detected at 2.7% for T54S, 1.5% for V36L, and <1% for V36M, T54A, and R155K). The clinical relevance of the presence of these variants as a dominant quasispecies prior to treatment remains unclear; however, their presence did not always preclude treatment success in telaprevir clinical trials. Response appeared to be unaffected in treatment-naïve patients, however, the presence of baseline variants may impact the response to T/PR in certain patient populations, such as prior null responders. Indeed, other factors such as response to peginterferon and ribavirin [30] as well as adherence to the treatment regimen likely play a larger role in the response and ultimate clinical outcome to the telaprevir treatment regimen.

### Resistance Profiles in Patients Who Did Not Achieve an SVR

The majority of patients who did not achieve an SVR had detectable resistant variants at the time of treatment failure. According to prior treatment history, the proportion of non-SVR patients with detectable resistant variants at the time of failure was 79% (44/56) of treatment-naïve (T12PR, ADVANCE), 69% (20/29) of prior PR relapsers, 61% (23/38) of prior PR partial responders, and 81% (75/93) of prior PR null responders (REALIZE, T12PR48 arms).

#### On-treatment Virologic Failure

On-treatment virologic failure rates during the telaprevir treatment phase of the first 12 weeks of treatment were low in both treatment-naïve and prior relapsers, and higher in prior non-responders, emphasizing the importance of responsiveness to PR in preventing virologic failure. Irrespective of prior response, virologic failure during the telaprevir treatment phase was associated with higher-level telaprevir-resistant variants, indicating that the telaprevir-based regimen suppressed wild-type virus and lower-level variants in most patients. Together, these data suggest that virologic failure is primarily due to an insufficient PR response and failure of PR to inhibit higher-level telaprevir-resistant variants. Interestingly, SVR rates in the nonresponder population were significantly increased by the addition of telaprevir to PR alone [31], suggesting that in some of these patients, the responsiveness to PR which may have been in part restored by telaprevir was sufficient to suppress the selection of resistance. In addition, virologic failure was more common in genotype 1a patients than in genotype 1b patients, and was predominantly associated with the V36M+R155K variant. This is likely because each of the two mutations in this relatively fit, higher-level resistant variant, V36M+R155K, has a lower genetic barrier in subtype 1a than in subtype 1b.

On-treatment virologic failure after telaprevir treatment, during the PR treatment phase, was low in both treatment-naïve and treatment-failure populations, and was associated with higher-level resistant variants but also with a large number of lower-level resistant variants and wild-type virus. These results suggest that the 12-week telaprevir-based regimen was able to suppress wild-type and most of the resistant variants, preventing subsequent on-treatment virologic failure during PR treatment in the majority of patients.

#### Relapse

Relapse was likely due to replication of residual virus that remained below the limit of detection at the end of treatment. While viral sequencing at the time of relapse can provide insight into what virus was present at the time when treatment was stopped, because the viral population is no longer under selective pressure it can evolve after the end of telaprevir treatment prior to sequencing. As described further below, the lower fitness of the resistant variants probably resulted in their loss from the population and replacement with wild-type virus. This process accelerated after the end of treatment as replication increased to baseline levels. This may be one of the reasons why relapse, and to a lesser extent virologic failure during the PR phase, was associated with a higher percentage of lower-level resistant variants and wild-type virus than on-treatment virologic failure. Alternatively, it is possible that in some of these patients, lower-level resistant variants and wild-type virus were not completely eradicated by treatment.

### Optimizing the Telaprevir/Peg-IFN/RBV Combination Regimen to Maximize SVR

Clinical virology analyses from studies of telaprevir, peginterferon and ribavirin in treatment-naïve and treatment-failure patients with genotype 1 chronic hepatitis C have provided further insight into the optimal treatment regimen needed to maximize response rates and minimize resistance. These results show that in a telaprevir-based regimen, the primary role of telaprevir is to inhibit wild-type virus and variants with lower-levels of resistance to telaprevir (Figure 4). The complementary role of Peg-IFN and RBV is to clear any remaining telaprevir-resistant variants, especially higher-level telaprevir-resistant variants which pre-exist in all chronically infected patients, albeit at low frequencies. Based on this framework, a telaprevir-based treatment regimen should have a telaprevir dose and duration that results in clearance of wild-type HCV and lower-level telaprevir-resistant variants, and a PR duration sufficient to clear any remaining variants. Clinical virology data suggest that both the dose (750 mg q8h) and the duration (12 weeks) of telaprevir treatment and the duration of PR treatment (24 or 48 weeks) contribute to determining a successful treatment outcome for most patients.

#### Role of Telaprevir in the Combination Regimen

Telaprevir combination treatment resulted in a rapid elimination of wild-type virus, predominantly driven by telaprevir. Patients who discontinued treatment early (before 4 weeks) for non-virologic reasons had residual virus that was predominantly wild-type, showing that a longer duration of telaprevir treatment is required to completely clear wild-type virus. In most patients who received longer durations of treatment (over 8 weeks of telaprevir), the viral population consisted predominantly of telaprevir-resistant variants. Furthermore, in ADVANCE, the T8PR regimen resulted in a slightly higher on-treatment virologic failure rate during the PR treatment phase (7.4%) than in the T12PR group (3.9%); and a greater number in the T8PR group were associated with wild-type or lower-level telaprevir-resistant variants [8]. The increase in the proportion of lower-level telaprevir-resistant variants suggests that the T8PR treatment provides a lower selective pressure than the T12PR treatment, and that 8 weeks may not be sufficient for some patients to fully clear lower-level variants and prevent subsequent failure during PR treatment. These data show that 12 weeks of telaprevir cleared all virus in most patients, and consistently eradicated wild-type virus in all patients. Therefore, a telaprevir duration of 12 weeks increases the probability of SVR in most patients.

#### Role of PR in the Telaprevir Combination Regimen

After wild-type and lower-level telaprevir-resistant variants have been eradicated and only higher-level telaprevir-resistant variants remain, telaprevir has little further utility because of the higher-level resistance of the remaining variants to telaprevir. Thus, it is the primary role of PR to eradicate these higher-level resistant variants. Higher-level telaprevir-resistant variants have reduced fitness and exist at a low prevalence before treatment. Although the variants remain fully sensitive to PR treatment, patients have variable responses to PR treatment. Thus, the duration of PR treatment will depend on individual patient responsiveness. As a measure of responsiveness to PR, extended rapid virologic response (eRVR), defined as undetectable HCV RNA at 4 and 12 weeks of treatment, was a good predictor of SVR after a total of 24 weeks of treatment in treatment-naïve patients. The ILLUMINATE study demonstrated non-inferiority of 24- and 48-week treatment durations in patients achieving an eRVR [24]. In patients not achieving an eRVR or who had a prior nonresponse to PR, a longer duration of 48 weeks was assigned. Patients who completed the full treatment duration of 24 or 48 weeks had very low relapse rates and similar resistance profiles of mostly wild-type or lower-level telaprevir-resistant variants.

#### Evolution of Resistant Variants after Treatment

Telaprevir-resistant variants are less fit than wild-type virus, providing a mechanism by which variants may be replaced by wild-type virus over time in the absence of drug-selective pressure in patients. In a Phase 1 study of 14 day telaprevir dosing (Study 101; n = 24), wild-type virus began to reappear in some patients 10 days post-dosing and was predominant by 3 to 7 months post-dosing in almost all patients [10].

Sequencing results obtained after treatment from the follow-up period of Phase 3 trials in patients who did not achieve an SVR suggest that changes in the viral population over time favor the replacement of telaprevir resistant variants with wild-type virus. Kaplan-Meier modeling suggests that resistant variants were lost at a similar rate in treatment-naïve and treatment-experienced patients, but that resistant variants were lost significantly more rapidly in 1b patients than in 1a patients. As reported in Sullivan et al., this subtypic difference results from a difference in replicative fitness in vivo between the variants commonly associated with treatment failure in each subtype. This difference results in a median time to WT of about 10 months after treatment failure in 1a patients as compared to only about 1 month in 1b patients.

These results from Phase 3 studies are supported by an interim analysis of an on-going, three-year observational study (EXTEND) which showed that within a median follow-up time of about 2 years almost 90% (50/56) of patients no longer had resistance detectable by population sequencing. Furthermore, a more sensitive, clonal sequence analysis was performed in a representative subset of 20 patients who were wild-type by population sequencing. This analysis showed that viral populations at the follow-up time point were not significantly enriched in resistant variants compared to baseline for each patient [31]. These results were corroborated by another study of 13 patients from Phase 1 studies, which compared the frequency of resistant variants determined by clonal or ultra-deep sequencing at baseline and in a follow-up sample 4 years (on median) after treatment. Similarly to EXTEND, all patients returned to their pre-treatment state at the follow-up time point [32], [33].

Together, these data suggest that resistant variants are detectable by population sequencing in most patients who fail to achieve SVR, but that their levels decline over time after treatment, often to levels undetectable by population sequencing. Clonal sequencing analyses further indicate that the frequency of resistant populations eventually returns to levels similar to those before treatment. As resistance is not likely to be genetically archived as with HIV or HBV, it may be possible to retreat patients with alternative treatment options, even if they include a previously used drug or class of antiviral agents. Future studies in patients who have failed regimens containing telaprevir or other classes of antiviral agents will be critical in assessing this hypothesis.

## Supporting Information

Figure S1 **Location of HCV NS3/4A Protease Amino Acid Substitutions Conferring Decreased Sensitivity to Telaprevir.** Yellow ribbon represents the NS4A cofactor; blue amino acids represent the catalytic triad of the protease.
(TIF)
