# Supplementary figures and images for: Hepatitis C Viral Evolution in Genotype 1 Treatment-Naïve and Treatment-Experienced Patients Receiving Telaprevir-Based Therapy in Clinical Trials
Source: PLoS One. 2012 Apr 12;7(4):e34372. doi: 10.1371/journal.pone.0034372 (PMC3325239; doi:10.1371/journal.pone.0034372)

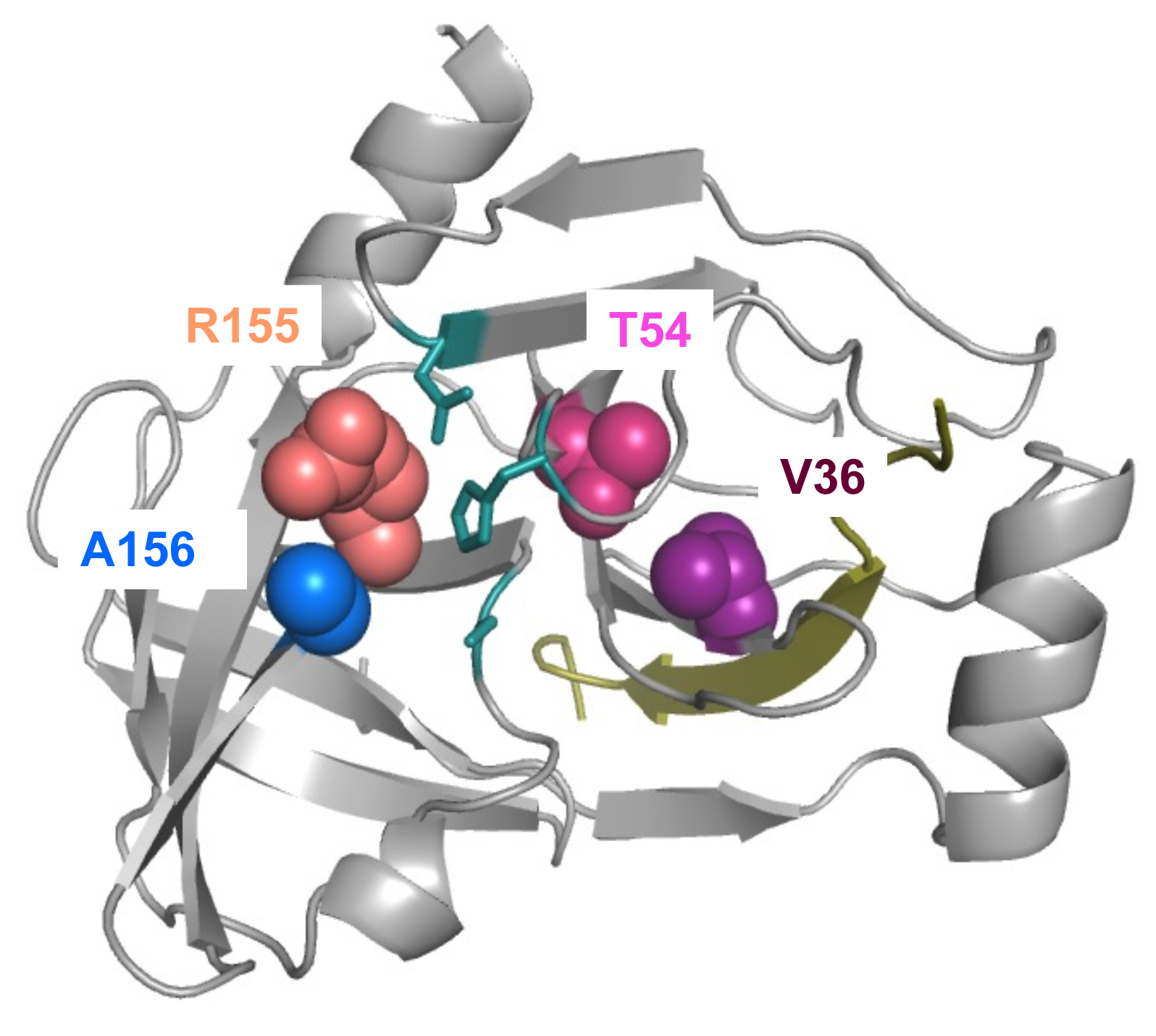

Supplement: Figure S1 — Location of HCV NS3/4A Protease Amino Acid Substitutions Conferring Decreased Sensitivity to Telaprevir. Yellow ribbon represents the NS4A cofactor; blue amino acids represent the catalytic triad of the protease. (TIF) [file pone.0034372.s001.tif]
